# Multi-week prediction of livestock chill conditions associated with the northwest Queensland floods of February 2019

**DOI:** 10.1038/s41598-022-09666-z

**Published:** 2022-04-08

**Authors:** Tim Cowan, Matthew C. Wheeler, Catherine de Burgh-Day, Hanh Nguyen, David Cobon

**Affiliations:** 1 Centre for Applied Climate Sciences, University of Southern Queensland, Toowoomba, Australia; 2 Bureau of Meteorology, Melbourne, Australia

**Keywords:** Atmospheric dynamics, Environmental impact, Natural hazards

## Abstract

The compound extreme weather event that impacted northern Queensland in February 2019 featured record-breaking rainfall, persistent high wind gusts and relatively cold day-time temperatures. This caused livestock losses numbering around 500,000 in the northwest Queensland Gulf region. In this study, we examine the livestock chill conditions associated with this week-long compound weather event and its potential for prediction from eleven world-leading sub-seasonal to seasonal (S2S) forecast systems. The livestock chill index combines daily rainfall, wind and surface temperature data. Averaged over the event week, the potential heat loss of livestock was in the moderate to high category, with severe conditions on the day of peak rainfall (5 February). Using calibrated forecasts from the Bureau of Meteorology's S2S forecast system, ACCESS-S1, a 1-week lead prediction showed a 20–30% probability of extreme livestock chill conditions over the northwest Queensland Gulf region, however the highest probabilities were located to the west of where the greatest livestock impacts were observed. Of the remaining ten S2S systems, around half predicted a more than 20% chance of extreme conditions, more than twice the climatological probability. It appears that the prediction accuracy arose from the skilful forecasts of extreme rainfall, as opposed to cold day-time temperature and strong wind forecasts. Despite a clear association between the observed extreme weather conditions and an active Madden–Julian Oscillation (MJO) event stalling in the western Pacific, the majority of 1-week lead S2S forecasts showed little indication of a slow-down in the MJO. As the livestock chill index was developed for southern Australian sheep, it may not be the best metric to represent the effects of exposure on tropical cattle breeds. Hence, this study draws attention to the need for tailored diagnostics that better represent the cold effects of summer tropical cyclones and tropical depressions on northern Australian livestock.

## Introduction

**Figure 1:**
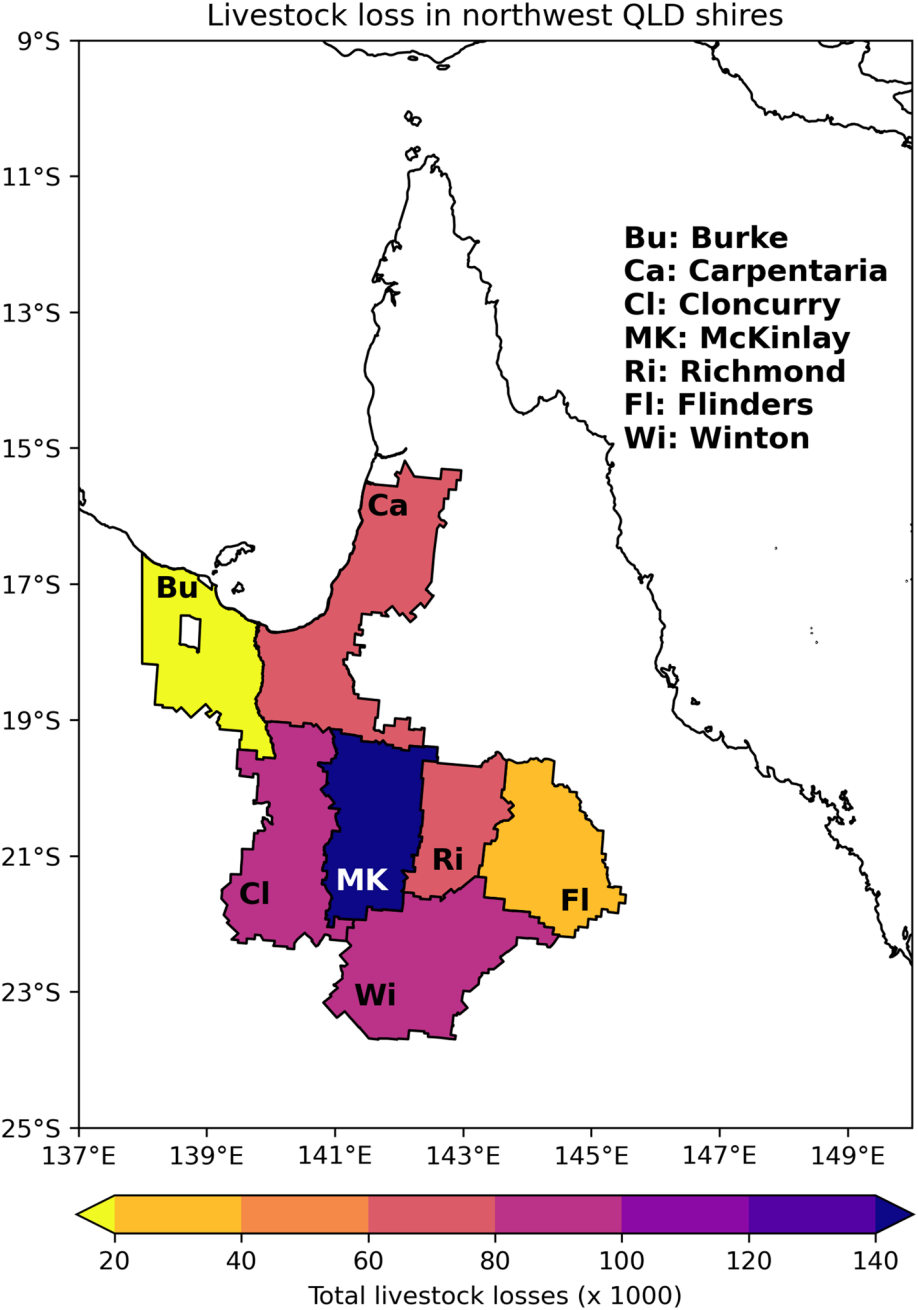
Total livestock losses during the February 2019 floods. The map shows the total livestock losses in the Shires of northwest Queensland during the early February floods. Livestock include cattle, sheep, horses and goats. The Burke Shire (Bu) reported no livestock loss, whilst the McKinlay Shire (MK) reported the highest losses, with 130,000 + cattle deaths. Data from: https://www.beefcentral.com/news/final-tally-reached-for-northwest-qlds-february-2019-flood-losses/.

The early February 2019 floods in northern Queensland, inland from the Gulf of Carpentaria (hereafter referred to as the Gulf), inundated an area close to 13 million hectares, causing wide-spread destruction of livestock^1^, pastures^2^, infrastructure^3^ and ultimately large economic costs to northern Queensland communities estimated at $5.68 billion AUD^4^. Although initial reports suggested cattle and sheep losses exceeded 620,000 and 48,000 respectively^1^, a more accurate count of livestock losses estimated 457,000 head of cattle and 43,000 sheep, with destruction of 22,000 km of fencing and 29,000 km of roads (https://www.beefcentral.com/news/final-tally-reached-for-northwest-qlds-february-2019-flood-losses/). The central northwest Queensland Shire of McKinlay reported the greatest livestock losses (region MK in Fig. 1) with deaths of over 132,000 cattle and 11,000 sheep. The adjacent Shires to the west (Cloncurry) and southeast (Winton) experienced livestock losses exceeding 86,000 and 97,000 respectively (regions Cl and Wi in Fig. 1). Winton Shire saw the highest loss of sheep with more than 26,000 head, making up 27% of the total livestock losses in that region. Of the inland regions, Flinders Shire, on the western edge of the Great Dividing Range reported the fewest deaths at over 25,000 head (region Fl in Fig. 1), while Burke Shire bordering the central Gulf coast reported no losses. Anecdotal evidence suggests that native fauna losses were much higher than livestock losses^2^. Even as the event was unfolding, media reports suggested that one of the main reasons for the unprecedented deaths was the exposure of drought-weakened livestock to relatively cold and wet conditions (https://www.beefcentral.com/news/cattle-losses-mount-as-northwest-qld-faces-monumental-flood-event/).

While the Temperature Humidity Index has been successfully used to quantify the heat stress in *Bos taurus* (cooler climate) and *Bos indicus* (warmer climate) cattle breeds^5^, there is a currently a knowledge gap in our understanding of the effect of cold stress on northern Australia cattle^6^. Less is known about cattle comfort during the Australian summer months (December to February) when temperatures can rapidly drop when a monsoon depression or tropical cyclone makes landfall, as was the case in February 2019 over the Gulf ^7^. Previous studies have predominantly focused on beef cattle in North America where animals have access to sheltered pens for protection from sub-zero temperatures^8,9^ or have focused on the detrimental impacts of warmer temperatures on northern Australia forage production and animal stress^10^. As noted in a cattle welfare assessment in feedlots from 2008^11^, heat loss in cattle is mostly driven by rain and wind, as opposed to cold air temperatures, with muddy conditions adding to the animal welfare risk^12^. Hence, one of the main motivations of this study is to quantify the observed evolution of the chill conditions of this extreme compound weather event experienced by northern Queensland livestock, including the factors that contributed to surface chill like heavy precipitation, relatively cold surface temperatures and wind. This is achieved using the livestock chill index (see “Data and methods”), although we later discuss its appropriateness for the northern tropics in the “Discussion”.

The weather system responsible for the extreme compound event was a quasi-stationary tropical low, described as a warm-air advection event (anticyclonic turning of winds with height) with cooler mid-level air drawn in from higher latitudes^7^. In the lead up to the tropical low deepening (26-27 January), a shallow inland trough stretched across tropical northern Australia, linking tropical cyclone Riley (off the northwest coast) and a tropical low over Cape York Peninsula^13^. The low responsible for the flooding formed a core on 30 January, and then deepened to 992 hPa over the next five days whilst remaining somewhat stationary inland from the Gulf^13,14^. At the same time, an active pulse of the Madden Julian Oscillation (MJO) stalled in the western Pacific^15^ for about 18 days, in contrast to the five days the MJO remained in Phases 6 and 7 in early January^1^. The stalling of the MJO is thought to have been a key driver of the event^1,15^. The near-stationary tropical low drove day-time temperature anomalies down to −10 °C (absolute temperatures of ~ 25 °C) at particular locations across the Cloncurry and McKinlay Shires, and wind gusts between 70 and 80 kph in the towns of Cloncurry, Julia Creek, and Winton^1^. In contrast to the drought and widespread heatwave conditions^16^ in December 2018 and January 2019, early February saw record-breaking rain and flooding in the region, fed by strong onshore easterly flow south of the monsoon trough^13^.

After the event, a study found that the Bureau of Meteorology's sub-seasonal to seasonal (S2S) forecast system, Australian Community Climate and Earth-System Simulator—Seasonal Version 1 (ACCESS-S1), successfully forecast a more than doubling of the climatological likelihood of extreme rainfall, winds and cold temperature anomalies for the week of 31 January–6 February, from a 24 January forecast^1^ (i.e. the lead week 1 prediction). However, the lead week 2 forecast from 17 January was far less skilful. Limitations on the prediction skill of the event's extreme rainfall beyond 1 week was also seen in a suite of ensemble numerical model forecasts from the UK Met Office^14^, with prediction errors generated locally in a lead week 1 forecast with convective rainfall overestimated along the western Cape York coast. This was further supported in a study focusing on a subset of four S2S models^15^, which suggested that three of the models could only predict an extreme rainfall event (off the coast of Townsville in far northeast Queensland) at a higher percentile ranking than their hindcast median, at a lead time of just over 1 week. The fourth model, on the other hand, showed good rainfall prediction skill beyond 2 weeks, which the authors argued to  result from the small ensemble size (*N* = 4) limiting the robustness of the statistical significance. While previous studies focused on the rainfall prediction of the flood event^1^, and the nature of atmospheric circulation^7,14^, here we focus on quantifying the livestock chill conditions of this event, including the observed evolution over late January and early February 2019, and the prediction of these conditions in the S2S models and ACCESS-S1. Given the compound nature of the extreme event, our main motivation here is to verify whether the livestock chill index, which combines rainfall, temperature and wind, could be better predicted than the individual variables, using the 2019 flood as a case study. We also conduct an evaluation of the forecasted trajectory of the MJO during the event to explore if the stalling was well-predicted and whether S2S models that more skilfully predicted the extreme conditions similarly predicted the MJO to stall. This study provides an opportunity to evaluate the performance of ACCESS-S1 (noting its output is calibrated against observations) against the world's best seasonal prediction models. Later in the study, we discuss the validity of using the livestock chill index for this event and outline a possible alternative that may better represent the conditions experienced by the tropical cattle breeds in flood events.

## Results

### Observed chill conditions

**Figure 2:**
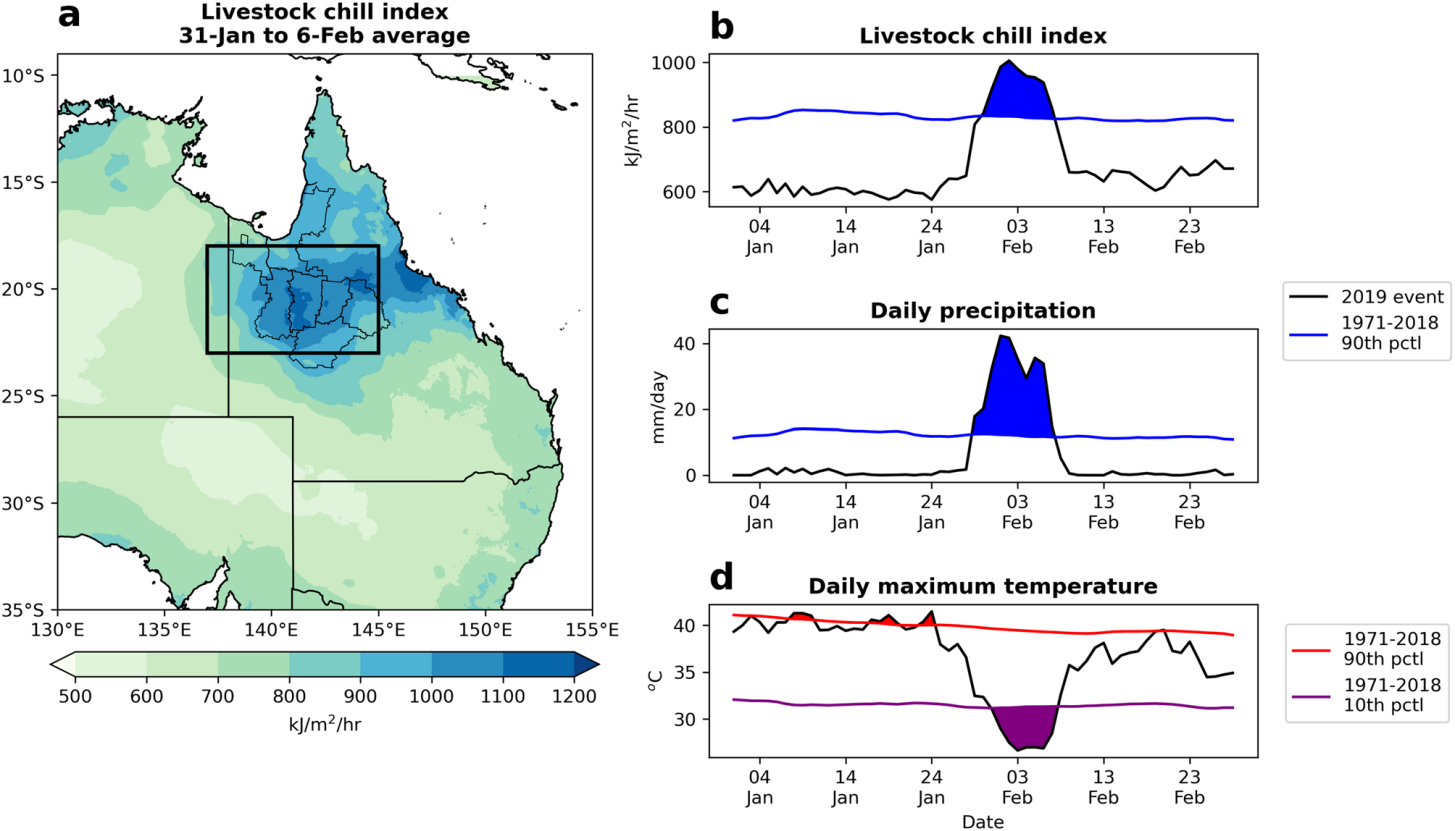
Average livestock chill conditions during the February 2019 floods. (**a**) Spatial pattern of the livestock chill index averaged over 31 January to 6 February 2019. (**b**) Area averaged livestock chill index (black line) over the Gulf region (rectangular region, 137–145° E, 18–23° S) from 1 January to 28 February, with a multi-year daily 90th percentile for 1971–2018 (blue line) and days above the 90th percentile (shading). (**c**,**d**) As in (**b**) but for  (**c**) daily precipitation and (**d**) daily maximum temperature, with the 90th (red line) and 10th (purple line) percentiles. The method for calculating the multi-year daily percentile is detailed in the Data and methods. The Gulf region represents the area with the highest livestock losses during the week-long event (Fig. 1).

The average livestock chill values during the peak flood week (31 January–6 February) are shown in Fig. 2a, highlighting the extreme nature of the cold and wet conditions stretching from Cloncurry Shire to the greater Townsville area (146.8° E, 19.3° S) along the northeast Queensland coast. The regions that experienced the chilliest conditions, with heat losses exceeding 1100 kJ/m^2^/h, included Townsville, the northern edge of the Flinders Shire and the southwest part of McKinlay Shire. It is no coincidence then that McKinlay Shire bore the brunt of the livestock losses, almost double the losses of the Cloncurry Shire with the second highest count (Fig. 1). For the flooded inland regions, the livestock chill index surpassed 1000 kJ/m^2^/h on 29 January, peaking over the inland Gulf region (region within box, Fig. 2a) on 2 February with a daily average heat loss of 1005 kJ/m^2^/h (Suppl. Fig. 1f.). On the same day, the town of Cloncurry (140.51° E, 20.67° S) broke its record for the highest daily rainfall in February^1^ at 178.2 mm and experienced wind gusts of 80 kph. The highest grid-point scale chill conditions of 1221 kJ/m^2^/h occurred in the southern inland Gulf region on 5 February near the town of Winton (Suppl. Fig. 1i), putting this location well into the severe chill category. This same date saw a record-breaking 233 mm of rain in the town of Julia Creek and gusts of 70 kph in Winton^1,13^. By 8 February the quasi-stationary tropical low had weakened considerably and moved out to the Coral Sea, leading to a reprieve in chill conditions across northwest Queensland (Suppl. Fig. 1).

The temporal evolution of the livestock chill index, averaged over the inland Gulf region, shows that the extreme conditions (i.e., above the multi-year daily 90th percentile) lasted 9 days from 30 January to 7 February (Fig. 2b). Here the 90th percentile lies above 800 kJ/m^2^/h. Similar to the chill conditions, the observed rainfall over the inland Gulf region surpassed the extreme threshold (daily 90th percentile) for 10 consecutive days from 29 January to 7 February, with two prominent peaks on 2 and 5 February (Fig. 2c). These two rain peaks helped maintain the severe chill conditions in that four-day period. Slightly lagging the rainfall, the daily maximum temperatures fell below the daily 10th percentile on 31 January and remained relatively cold until 7 February (Fig. 2d). One suggested factor in the high livestock loss was the rapid fall in day-time temperatures from above 40 °C in mid-January to the low-mid 20 °C temperatures during the flood, on the back of heatwaves in November and December^16,17^ and long-term drought^2^. This rapid drop from extreme hot to extreme cold day-time temperatures occurred over a relatively short period of one week (Fig. 2d), with anecdotal observations from beef producers of a possible contribution to cattle losses^1^.

### Multi-week prediction of livestock chill conditions

**Figure 3:**
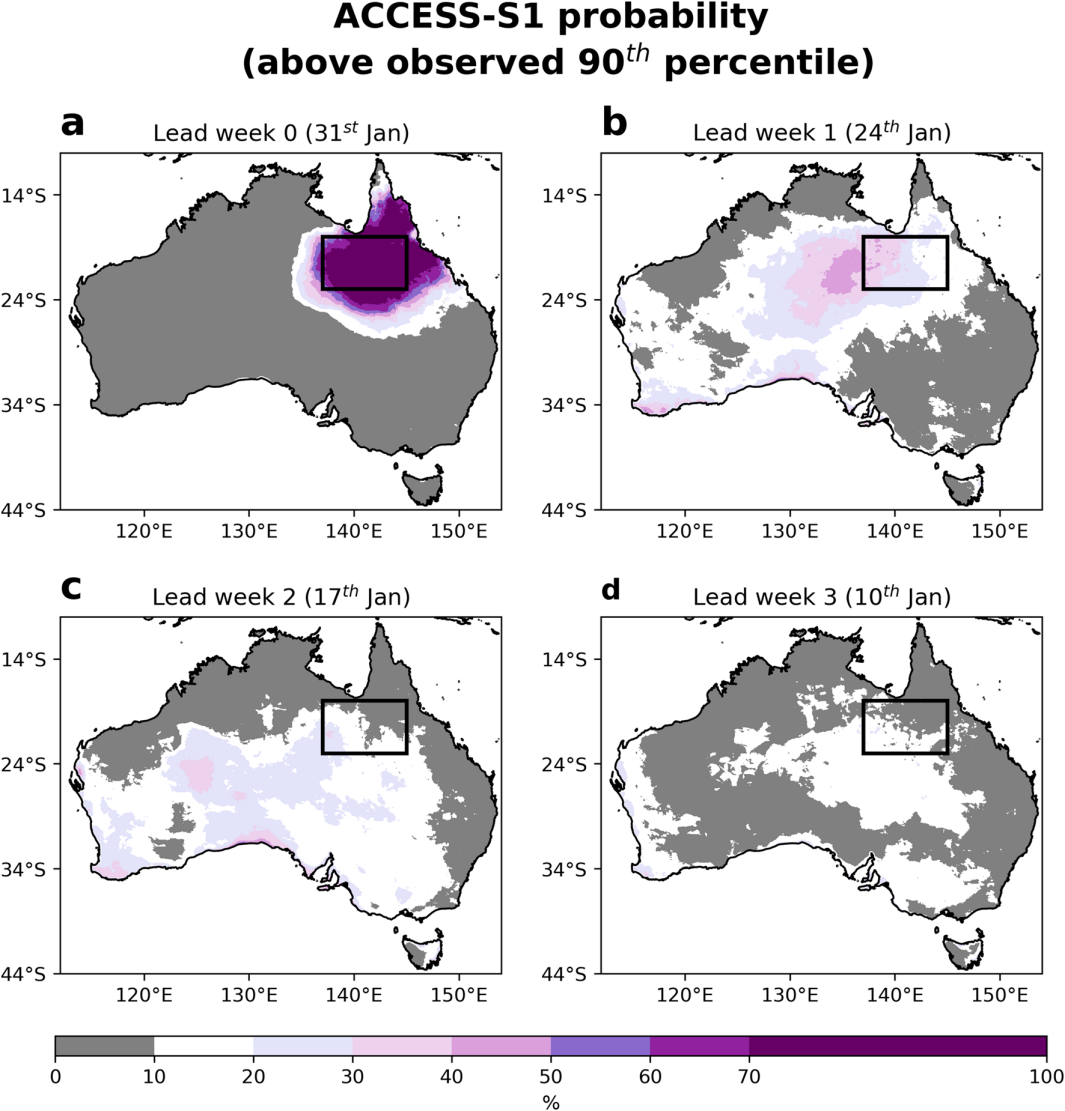
Lead week 0–3 forecasts of the chance of extreme livestock chill conditions during the February 2019 floods from ACCESS-S1. Shown are the ACCESS-S1 forecasts of the livestock chill index being above the observed multi-year (1971–2018) daily 90th percentile, targeted to the week of 31 January– 6 February, for (**a**) lead week 0; 31 January, (**b**) lead week 1; 24 January, (**c**) lead week 2; 17 January and (**d**) lead week 3; 17 January. The Gulf region is shown by the black rectangle.

As discussed in recent prediction studies on this event^1,14^, ACCESS-S1's lead week 1 forecast showed a 40–70% probability (i.e., more than double the climatology) of the inland Gulf region experiencing quintile 5 rainfall and quintile 1 day-time temperatures during the peak of the February 2019 floods. We extend this further to look at the ACCESS-S1 forecasts of potential extreme (above the observed 90th percentile) livestock chill conditions from a lead week 0 (31 January) to week 3 (10 January) initialisation, noting the lagged ensemble approach (see Data and methods). It is clear that the lead week 0 forecast successfully predicts the extreme chill over the peak flood week with probabilities between 70 and 100% (Fig. 3a). This is expected as ACCESS-S1 was initialised between 29 and 31 January and severe chill conditions were already being observed on 30 January. Turning to the lead week 1 forecast, the majority of the inland Gulf region show probabilities of between 20 and 40%, increasing from east to west (Fig. 3b). Some isolated locations show probabilities of above 40%, although the main bulls-eye centre lies in the southern Northern Territory (between Alice Springs and the Queensland/Northern Territory border). The lead week 1 probabilities in the inland Gulf region represent between 2 and 4 times the climatological reference probability of 10%, consistent with rainfall, temperature and wind forecasts^1^. Moving back to lead week 2 and 3 forecasts (Fig. 3c,d), ACCESS-S1 shows a near climatological probability forecast, with likelihoods of extreme conditions slightly above 10% in the southern Gulf region.

**Figure 4:**
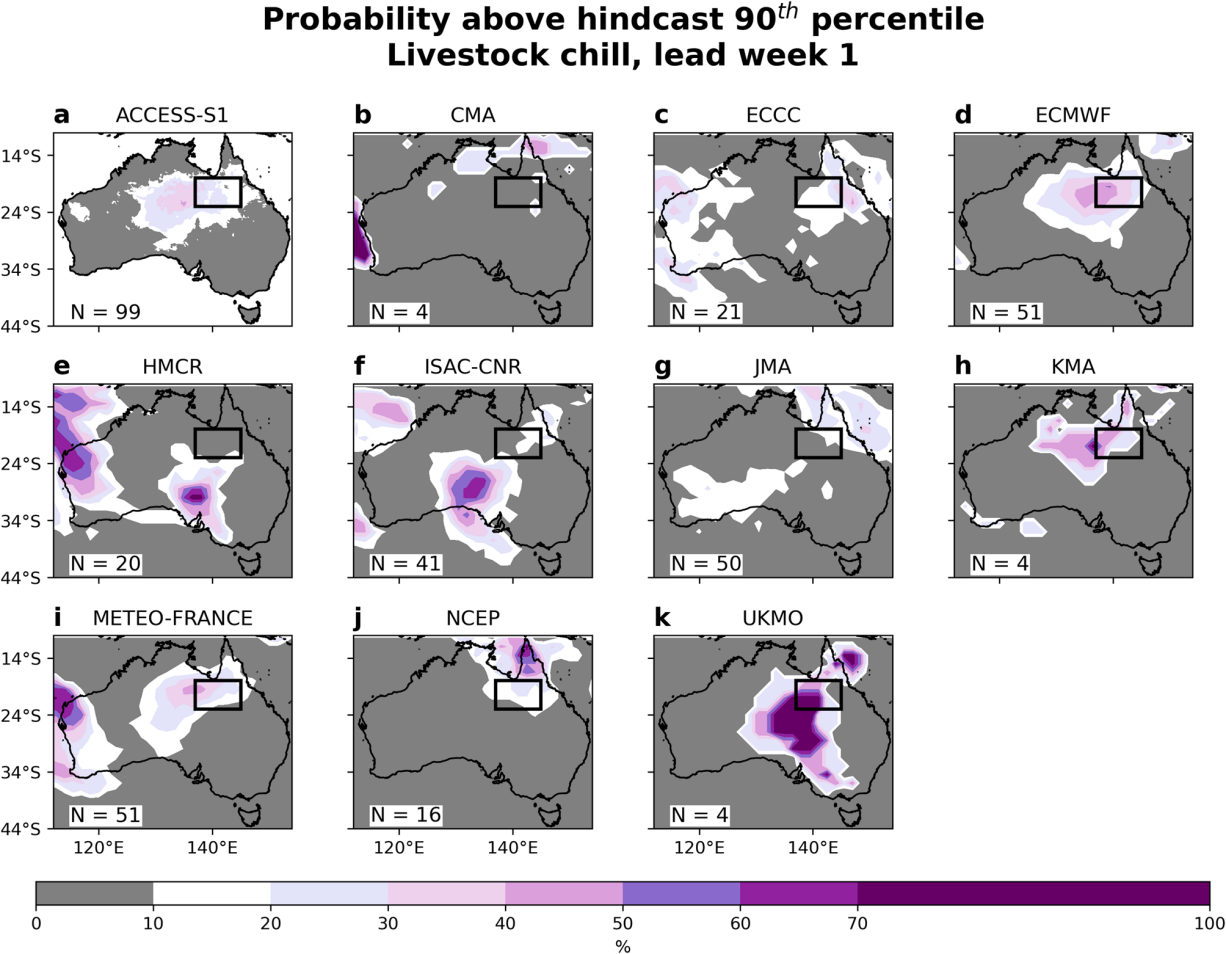
Lead week 1 forecast of the chances for extreme livestock chill conditions from ACCESS-S1 and ten S2S forecast systems. Shown are the model forecasts of livestock chill index being above their respective hindcast multi-year daily 90th percentile, targeted to the week of 31 January to 6 February 2019, for (**a**) ACCESS-S1, (**b**) CMA, (**c**) ECCC, (**d**) ECMWF, (**e**) HMCR, (**f**) ISAC-CNR, (**g**) JMA, (**h**) KMA, (**i**) Météo-France (CNRM), (**j**) NCEP and (**k**) UKMO. Each model's hindcast years are listed in the Table 1, and the ensemble forecast size is shown in the bottom left corner of the panels.

Focusing on the lead week 1 forecast, we next assess the ten remaining S2S models and compare their forecasts to ACCESS-S1. Compared to the observed or hindcast extreme conditions, ACCESS-S1's forecast is similar over the Gulf region (comparing Figs. 3c and 4a). Unlike ACCESS-S1, the S2S model forecasts are not calibrated against observations^18^, and, as such, we focus on the forecast probability above each model's own hindcast 90th percentile; this takes into account implicit model biases which are present in both forecasts and hindcasts. That way, there is less emphasis on unrealistic forecast probabilities, as seen in a number of S2S lead week 1 forecasts in the context of what is considered extreme in observations (Suppl. Fig. 2). Of the ten S2S models, four show probabilities of extreme chill conditions of less than 10% for a majority proportion of the inland Gulf region (Fig. 4b,e,f,g). Similar to ACCESS-S1, another four model forecasts show probabilities above 20%, and place the chill maxima to the west and south of the impacted regions (Fig. 4d,h,i,k). The remaining two models forecast the extreme conditions predominantly along the far northeast coast, part of which encroaches into the Gulf region (Fig. 4c,j). From this model subset, the UKMO  model forecast shows the highest probabilities, exceeding 70%, although it places the main predicted impact region over southern Australia (Fig. 4k), coinciding with forecasts of extreme rainfall and cold day-time temperatures there (Suppl. Figs. 3k and 4k, respectively). It is likely, however, that these high probabilities partly stem from an incomplete sampling of possible extreme events calling in the robustness of the statistical significance, given the small number of available forecasts (*N* = 4) and hindcast (*N* = 7) members from the UKMO model and a relatively short hindcast period of 24 years. Two of the models, ECMWF and Météo-France, with 51 forecasts in their ensemble, show similar probabilities to ACCESS-S1, displaying a 30–40% chance of extreme chill over the western inland Gulf region (Fig. 4d,i), consistent with their rainfall, temperature and wind forecasts (Suppl. Figs. 3–5d,i). Based on the extreme wind forecasts, both ACCESS-S1 and ECWMF show greater than 20% probabilities (of the models with large ensembles). The lack of any extreme wind forecasts in other S2S models (Suppl. Fig. 5) suggests that for the models that predicted the extreme chill conditions, much of this signal was emerging from their extreme rainfall forecasts (Suppl. Fig. 3). A further commonality between some forecast ensembles is the placement of extreme chill probabilities too far north over the Cape York Peninsula (e.g., CMA, ECCC, JMA and NCEP). This might reflect the models forecasting the tropical low migrating eastward too rapidly, as seen in UKMO numerical model forecasts^14^. It is possible that the different hindcast periods across the S2S models present a challenge in comparing the extreme probabilities between models; however, we repeated the analysis using a common hindcast period (1999–2010) and the probability patterns remain largely unchanged (figure not shown). As detailed in recent studies, the week-long weather event that led to the extreme chill was associated with a stalling of the convective phase of the MJO over the western Pacific for ~ 10 days^1,15^. We next investigate whether the stalling of the MJO was predictable in the S2S models based on lead week 1 forecasts of the MJO.

### S2S prediction of the stalled MJO

**Figure 5:**
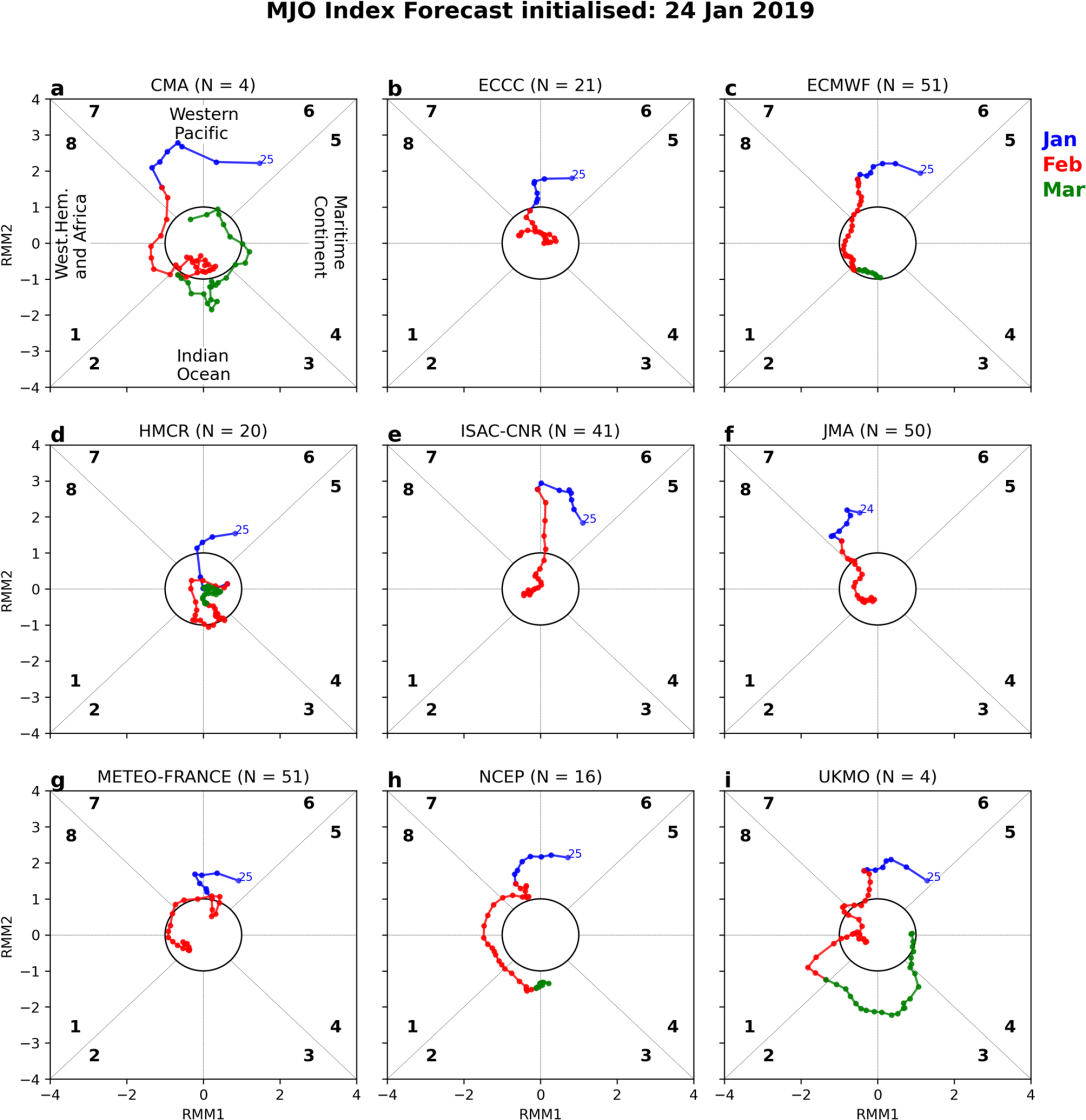
Time evolution of multi-member ensemble MJO predictions for nine S2S models, initialised on 24 January 2019 (lead week 1 forecast). Shown are the evolutions from late January (blue) through February (red) into March (green) for (**a**) CMA, (**b**) ECCC, (**c**) ECMWF, (**d**) HMCR, (**e**) ISAC-CNR, (**f**) JMA, (**g**) Météo-France (CNRM), (**h**) NCEP and (**i**) UKMO. Note, JMA is initialised on 23 January, hence its first date is 24 January. Despite not all the MJO forecasts extending to March, all cover the early February flood event. The observed MJO trajectory is shown in Suppl. Fig. 6g.

Ensemble mean MJO index predictions from nine S2S models (noting KMA did not supply MJO forecasts) for late January through February, initialised on 24 January (lead week 1), is shown in Fig. 5, noting that ensemble sizes that contribute to the means range from 4 (UKMO, CMA) to 51 (ECMWF, Météo-France). The observed evolution, from 25 January to early March, clearly shows the active MJO pulse shifting back and forth from phases 6–7 in the phase space diagram (Suppl. Fig. 6g). Determined from composites of weekly rainfall probabilities going back to the mid-1970s, when an active MJO pulse lies in phase 6 in the austral summer, there is a greater than 50% chance of upper tercile rainfall across northern Australia, falling to below 40% in phase 7 over the far northeast^19^. It is clear from the lead week 1 MJO predictions from the nine S2S models that nearly all fail to predict any substantial stalling in phases 6 and 7. The model ensemble forecasts from CMA, ECCC, HMCR and JMA show either the MJO becoming weak in February (inside the MJO phase diagram's unit circle) or moving into the western hemisphere (Fig. 5a,b,d,f). For models with the highest wind chill (and rain/temperature) probabilities in their lead week 1 forecast (ECMWF, Météo-France, NCEP and UKMO), there is a slight indication of a slow-down in the MJO through phase 7. The ECMWF and UKMO predictions show the MJO tracking in phase 7 during the first 8–10 days of February (Fig. 5c,f, respectively), while there is a clear folding structure in the MJO evolution of the Météo-France and NCEP (Fig. 5g,h, respectively). This infers that some of the models were able to partly capture the increase in MJO residence time over the western Pacific. One possibility is that a small subset of model members predicted the stalling MJO, however by averaging over larger ensembles, this acted to smooth out the signal. To test this, we repeated the analysis, only focusing on ensemble member forecasts where the livestock chill forecast exceeded the 90th percentile hindcast values in more than 60% of the inland Gulf region. The results show that the majority of these model ensembles do not show a more well-defined stalling structure in their MJO phase diagrams associated with the extreme chill conditions (i.e., one that is clearly different from using all model members), except for NCEP where there is a noteworthy stalling in the MJO evolution from phases 7 to 6 and back to 7 in the first 10 days of February (Suppl. Fig. 6e).

**Figure 6:**
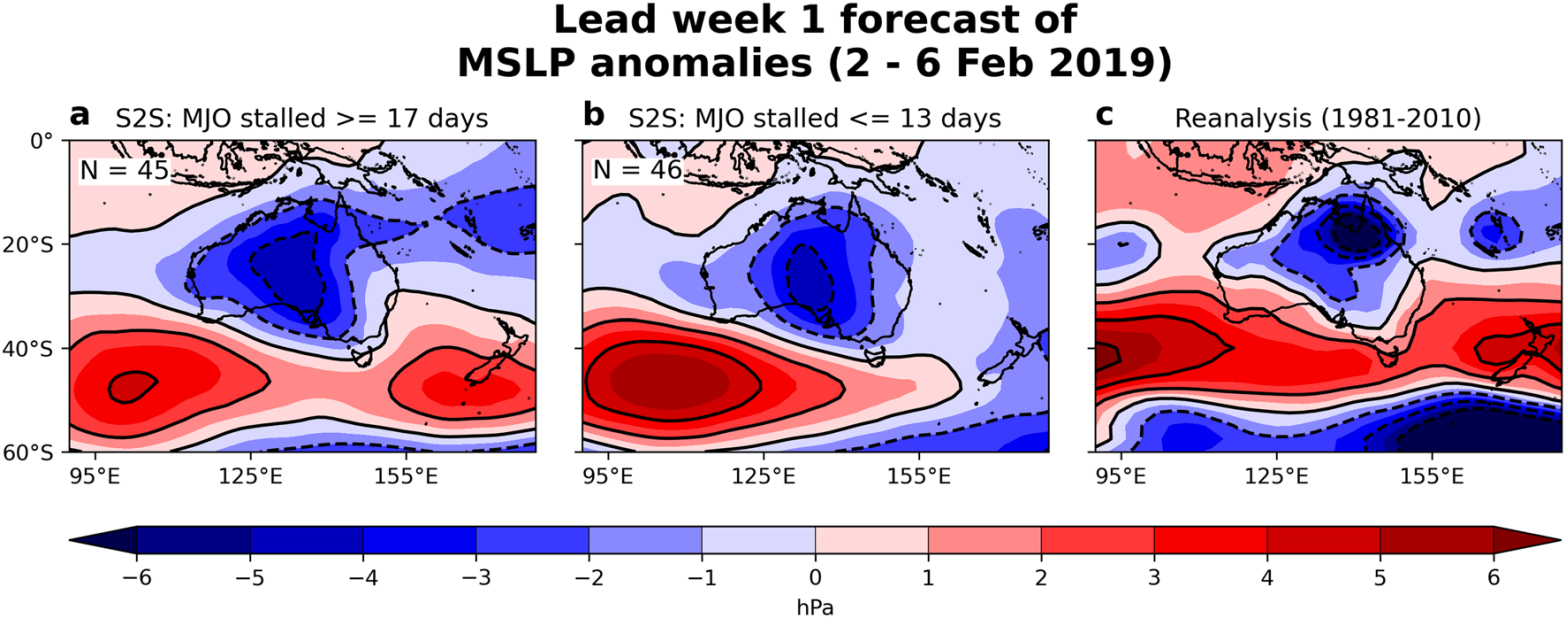
A comparison of daily MSLP anomalies averaged over the 2–6 February 2019 from reanalysis and lead week 1 forecasts from S2S multi-model experiment ensembles. Shown are the weighted multi-model ensemble average of lead week 1 forecasts of daily MSLP anomalies from six S2S models (ECCC, ECMWF, ISAC-CNR, JMA, Météo-France, NCEP) where individual member predictions showed (**a**) an active MJO pulse to stall for at least 17 days in phases 6 and 7; and (**b**) an active MJO pulse to stall for less than 13 days in phases 6 and 7. (**c**) NCEP reanalysis average for 2–6 February 2019 for the period 1981–2010. Each individual S2S forecast is referenced to its own hindcast climatology, with both the reanalysis and hindcast climatology smoothed using an 11-day running mean.

Turning our approach around, we focus on model ensemble members that most accurately predicted the stalled MJO in their lead week 1 forecast, to determine if they also predicted a more realistic broad-scale surface circulation (e.g., mean sea level pressure [MSLP] anomalies) during the extreme event. As such, two model subsets are created, one containing S2S members that predicted the active MJO to remain within phases 6 and 7 for at least 17 days, and the other with S2S members that predicted the MJO to pass through the same phases in less than 13 days. The first subset represents model members that predicted the MJO stall (i.e., the observed MJO stalled in phases 6 and 7 for 18 days), while the second subset represents model members that underpredicted the stalling MJO. The near-equal sized subsets (*N* = 45, 46) are created from six S2S models contain members in their ensemble suite that predicted and underpredicted the MJO slow-down (i.e., we excluded CMA, HMCR, UKMO). From this, the weighted multi-member averages of daily MSLP anomalies associated with these subsets are calculated. Compared to the reanalysis pattern, which shows the tropical low centred over the Gulf region and the southward-shifted subtropical ridge over southern Australia (Fig. 6c), the MJO stalled S2S average places the much broader anomalous depression too far southwest over central Australia. Yet the MJO stalled average skilfully predicts the monsoon trough to the east and the southern ridge (Fig. 6a). While the underpredicted MJO S2S average also simulates a broad weak depression over the southwest (Fig. 6b), it struggles to forecast the eastward extensions of the monsoon trough and southeast portion of the subtropical ridge, both seen in the reanalysis. This analysis suggests that S2S models that accurately depicted the MJO to stall in late January/early February also better reproduced the broad-scale circulation in the tropics and midlatitudes. On the other hand, there was a tendency for S2S models to place the tropical low too far south, consistent with the predicted impacts not centred over the Gulf region. Yet it remains to be seen what role the stalling MJO had on the extreme event, as to whether it was a cause or a symptom of the atmospheric conditions to the north of Australia.

## Discussion

This study has highlighted the extreme nature of the weather conditions during the February 2019 floods over northern Queensland, which devastated the livestock sector in many of the state's northwest Shires. Despite not yet being available to the general public at the time of the floods, forecasts of the livestock chill conditions from ACCESS-S1 a week prior to the start of the floods suggested the probabilities of extreme conditions at around 40% or ~ 4 times the climatological likelihood. This was supported by a number of international S2S forecast models. Across the models, it is clear that those with the highest probabilities of extreme chill conditions in their lead week 1 forecasts also predicted extreme rainfall and cold-daytime temperatures (relative to their own hindcasts). On closer inspection, it also appears that a forecast of extreme chill conditions does not necessarily mean a model is forecasting extreme winds and/or cool temperatures, as shown by the KMA, HMCR and ISAC-CNR models, noting the latter two models placed the chill 'bullseye' for a lead week 1 forecast too far southwest. Yet, if models are predicting extreme rainfall, temperatures and winds, then the expectation is a confident forecast of extreme chill conditions. Hence, there is value is having a livestock chill forecast, as opposed to relying on individual variable forecasts that may not capture the severity and compound conditions of a weather event.

Even though observations indicated the strong coincidence of the MJO stalling in phases 6 and 7 and the quasi-stationary behaviour of the tropical low responsible for the extreme conditions, only about four of nine sampled model ensembles forecast a slowdown of the MJO trajectory 1 week before the extreme event; however, no models forecast the MJO stalling to the same extent as was observed (~ 18 days). What is apparent is that, in general, models that performed better at predicting the MJO stalling better captured the monsoon trough and broad pressure ridge to the south of Australia, despite not accurately predicting the position of the tropical low. This raises the question as to why most S2S models seemingly struggled to forecast the MJO evolution, given these same models show good MJO skill out almost to 4 weeks^20,21^. Well recognised issues with MJO prediction skill in S2S models include faster decay of its amplitude from initialisation compared to observations, underestimation of MJO propagation across the Maritime Continent, and varied phase-dependent prediction skill^22^. Even  models with good MJO prediction skill, such as ACCESS-S1, have difficulty depicting the teleconnection of a strong MJO pulse on austral summer rainfall over northern Australia^21^. Typically, when the MJO is in phases 6 and 7, there is an increased probability of upper tercile rainfall over the far northern tropics of Australia, however this does not extend down into the inland Gulf region of Queensland^19^. While prediction skill in S2S models can be slightly extended by correcting biases^23^, further work is required in improving both the simulated teleconnection of the MJO with northern Australian weather conditions^21^ and more generally, the predictive performance for extreme rainfall in austral summer^24^. Underscoring the need for these improvements are the long-term observations showing a general slow-down in the MJO over the Maritime Continent, related to a strong warming of the Indo-Pacific warm pool, and projections showing a continuation of this warming^25^.

What is clear from the February 2019 event is that the week-long chill conditions, brought on several days of heavy rainfall, relatively cold maximum temperatures and sustained strong winds took a heavy toll on livestock, predominantly cattle. Specifically developed as a sheep graziers' tool for southern producers during the winter months, the livestock chill index was not designed for cattle, particularly northern tropical breeds. One area of future development in forecast prototypes is through tailoring a chill index for beef cattle that represents the conditions that animals experience in times of heat stress and cold snaps (i.e., as experienced from late 2018 into February 2019). On such example is the comprehensive climate index (CCI), which factors in the influence of surface temperature, relative humidity, solar radiation and wind speed^26^. The benefits of using an index like CCI over other widely used indices like the temperature-humidity index is the addition of environmental factors like wind and solar exposure^27^. Yet one issue with the CCI is that it does not factor in the apparent temperature response of an animal's wet coat, so it is perhaps more suitable for dry cooler winter extremes in southern Queensland. Other factors not considered in the CCI are the overall animal condition, their age, sex and breed, as well as their energy expenditure traversing boggy grounds. The CCI has been tested for cold extremes based on weather conditions in far cooler climates of midwest North America^26^ where temperatures regularly drop below 0 °C. As such, for tropical northern Australia, the CCI is likely a more appropriate tool to test if there is a high risk of animal mortality due to heat extremes, particularly in exposed open areas.

**Figure 7:**
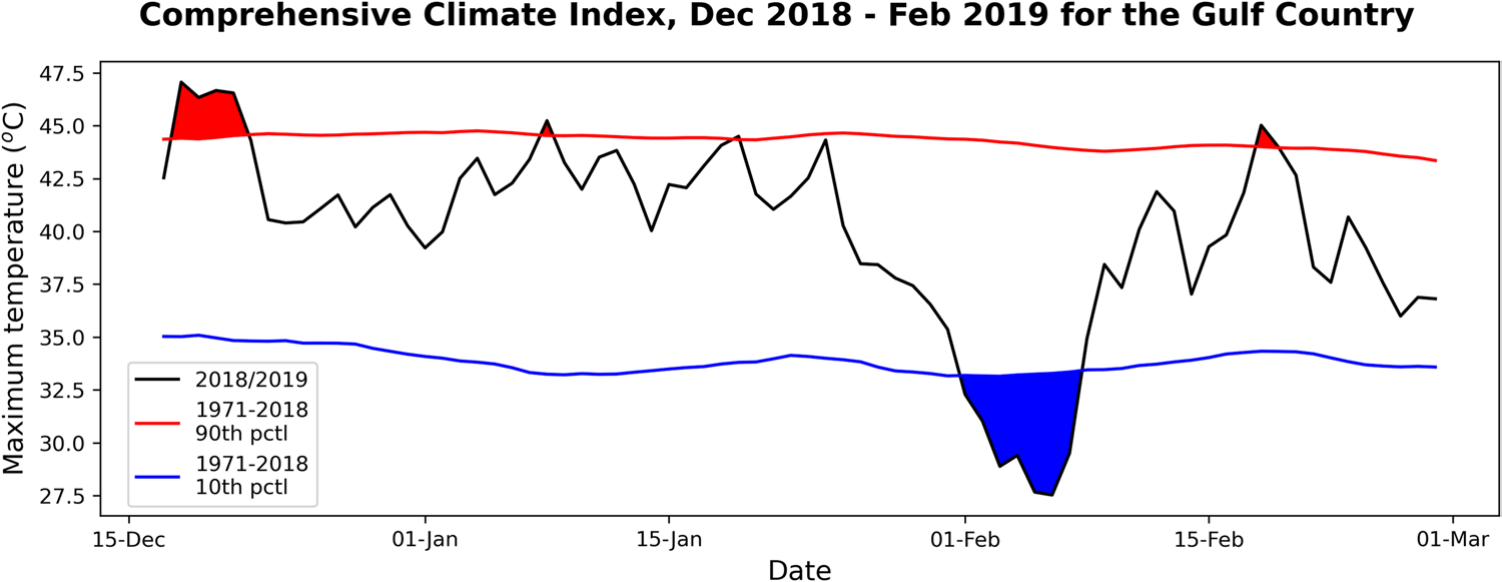
Comprehensive climate index over the inland Gulf region in late 2018/early 2019. Area-averaged comprehensive climate index (black line) over the Gulf region from late December 2018 to late February 2019, with a multi-year daily 10th and 90th percentile for 1971–2018 (blue and red lines, respectively) and days below/above the percentile (blue/red shading). The index has been calculated using maximum daily temperatures, daily maximum relative humidity, daily averaged wind speed, and daily-averaged solar radiation using the adjustment factors and equations listed in Mader et al.^26^.

The CCI has not been tested for cold and wet extreme conditions commonly seen during tropical storms and cyclones. In assessing the risk of cattle mortality in extreme events like the 2019 floods the CCI can be utilised in a similar way to the livestock chill index, by comparing daily CCI values to long-term multi-year daily percentiles, instead of arbitrary thresholds^26^. Analysing the CCI for the 2019 floods, it is clear that the index falls 6–7 °C below the multi-year daily 10th percentile, averaged over the inland Gulf region in early February (based on maximum temperatures; see Fig. 7), supporting the livestock chill values. The heat wave conditions are also captured by the CCI^16^ in mid-late December of 2018. Further analysis of CCI values determined from daily weather station observations over the affected region during the flood event suggests chill conditions were well below the daily maximum temperatures and apparent temperatures (not shown), but not in the range of the thermal stress thresholds determined by Mader et al.^26^. Hence, future work should be directed towards ground-truthing the impact of extreme CCI conditions (i.e., above/below the tails of the distribution) and rapid changes in CCI (hot to cold) over short timeframes on cattle numbers across northern Australia, instead of introducing a set of arbitrary CCI thresholds. This research has shown that the peak livestock chill conditions over the inland Gulf region matched where the highest number of livestock deaths occurred during the 2019 floods. However, more generally, the livestock chill index has yet to be fully tested for tropical cattle breeds in northern Australia in extreme wet and cold conditions in the summer months in other years. As the western warm pool expands and the MJO life cycle changes as climate models project^25^ it is possible that extreme events like the 2019 floods may become more common in the future, meaning tailored forecast products for livestock will be crucial.

## Data and methods

### Livestock chill index

The livestock chill index used here is an empirical model that describes the potential heat loss in newborn lambs and newly-shorn sheep^28^. While not typically used to describe the chill effects on cattle, we note that newly-shorn sheep would have a closer thermal comfort level to cattle breeds used across tropical and semi-arid northern Australia, compared to breeds in the south, given their short coats. Currently, this livestock chill index is used operationally by the Bureau of Meteorology for their Sheep Grazier Alert^29^, particularly relevant for southern and eastern Australia as this is where sheep represent a high proportion of domestic livestock numbers, and mortality due to hypothermia is of frequent concern^30,31^. Our justification for using this index is that it is currently the only index made available as a forecast on the multi-week time scale, albeit as a prototype. Also, around 9% of the total livestock deaths in the 2019 event were sheep, meaning for the mostly southern Shires in Queensland's northwest (Fig. 1), this index is directly relevant for this event. Later, we discuss caveats related to the wind chill index and a possible alternative metric.

The livestock chill index is defined as:

$$C = \left( {11.7 + 3.1v^{0.5} } \right)\left( {40 - T} \right) + 481 + 418\left( {1 - e^{ - 0.04R} } \right)$$

where *C* is the potential heat loss (kJ/m^2^/h), *v* is the mean daily wind speed (m/s), *T* is the average daily temperature (°C) and *R* is the daily rainfall (mm). Observed gridded daily rainfall and temperatures on a 0.05° × 0.05° grid (~ 5 km) were taken from the Bureau's Australian Water Availability Project (AWAP^32^). Average daily 2-m wind observations from a network of Bureau weather stations were interpolated onto the same 5 km grid as for AWAP^33^. Given daily varying wind data is only available since 1975, wind input prior to 1975 is based on a constant climatology^34^ calculated over 1975–2017. To maximise our climatology period, we chose the base period to be 1971–2018. Based on a recent report into lamb survival in southwest Victoria, a livestock chill value exceeding 1000 kJ/m^2^/h is considered high, with Bureau forecasts above this threshold raising a Sheep Grazier Alert, while chill conditions over 1200 kJ/m^2^/h are deemed severe^35^. Results from a trial alerting sheep producers to forecasts of low, medium or high chill values at several Victorian weather stations^36^ showed sensitivity thresholds at some stations were above 1100 kJ/m^2^/h. Along with the Sheep Grazier Alert that is based on deterministic forecasts, the Bureau also uses ACCESS-S1 output to produce two prototype chill forecast products on the multi-week to multi-seasonal time frame: one showing the potential for extreme livestock chill conditions and the other, the mean number of chill index days. It is these latter prototype forecasts that are the focus in this paper.

### ACCESS-S1 and S2S models

ACCESS-S1 is the Bureau's current operational multi-week to seasonal prediction system^37^, replacing the previous system POAMA (Predictive Ocean Atmosphere Model for Australia) in August 2018. The atmospheric component of the model is at a N216 (~ 60 km) resolution, which is then calibrated to the AWAP 5 km grid over Australia using quantile–quantile matching approach^38^. More details on ACCESS-S1's ocean model, land surface model and its initial conditions are outlined in Hudson et al.^37^. The model has been instrumental in the development and improvement of forecast products, such as thermal stress on corals^39^, the Northern Rainfall Onset for northern livestock producers^40^, rainfall bursts, as well for as a suite of climate extremes including heat waves, cold waves, forest fire intensity index and livestock chill index that are available on a Forecast Viewing Tool for project stakeholders^41^. Here we examined ACCESS-S1 multi-week forecasts of the livestock chill conditions in the week of 31 January to 6 February from four start dates ranging from lead week 3 (10 January 2019) to lead week 0 (31 January). These forecasts are composed of a 99-member ensemble produced from 33 members per day over 3 days. We additionally utilise ACCESS-S1's hindcasts, generated for the period 1990–2012, to evaluate how the forecasts compare against the model's own "historical" thresholds (as well as the observed thresholds for an extreme event). The hindcast ensemble consists of 11 members per start date, with four start dates per month (1st, 9th, 17th, 25th) across the 23-year hindcast period. The lead week 1 and 2 forecasts (24th and 17th January) are matched to the 25th and 17th January initialised hindcasts.

The forecast and hindcast data from ten S2S models were accessed from the S2S Project Database^18^. For each model, we compared their livestock chill predictions to the S2S forecasts of rainfall, temperature and wind, to explore the factors that contributed to the high chill values of the compound event. We excluded the Bureau's POAMA model due to the lack of wind data, however we argue that it is unlikely to have shown more skilful forecasts than ACCESS-S1 based on its underestimation of total rainfall during the flood event^1^. The ten S2S models were provided to the S2S database on a common 1.5° × 1.5° grid^42^ and consisted of one control run and a set of perturbed runs (Table 1). It should be noted that the S2S model runs are not calibrated against any observational products^43^; this is left to individual users of the S2S database because of different choice of hindcast length and window period to undertake the calibration (F. Vitart, *pers. comms*.).

**Table 1:** Forecast and hindcast information from ten S2S models and ACCESS-S1.

Model (version)	Ensemble size	Forecast dates in Jan	Hindcast period	Hindcast size	Hindcast dates in Jan	Ocean, sea-ice coupling	MJO index
CMA (BCC-CPS-S2Sv2)	4	10, 17, 24, 31	1994–2014	4	10, 17, 24, 31	Y, Y	Y
ECCC (GEPS 5)	21	10, 17, 24, 31	1998–2017	4	10, 17, 24, 31	N, N	Y
ECMWF (CY43R1)	51	10, 17, 24, 31	1999–2018	11	10, 17, 24, 31	Y, N	Y
HMCR (RUMS)	20	10, 17, 24, 31	1985–2010	10	10, 17, 24, 31	N, N	Y
ISAC-CNR (GLOBO)	41	10, 17, 24, 31	1981–2010	5	11, 16, 21, 26, 31	N, N	Y
JMA (GEPS1701)	50	10, 17, 24, 31	1981–2012	5	10, 20, 31	N, N	Y
KMA (GloSea5-GC2)	4	9, 16, 23, 30	1991–2010	3	9, 17, 25	Y, Y	N
Météo-France (CNRM-CM 6.0)	51	10, 17, 24, 31	1993–2014	15	8, 15, 22	Y, Y	Y
NCEP (CFSv2)	16	10, 17, 24, 31	1999–2010	4	10, 17, 24, 31	Y, Y	Y
UKMO (GloSea5)	4	10, 17, 24, 31	1993–2016	7	9, 17, 25	Y, Y	Y
ACCESS (S1)	99	10, 17, 24, 31	1990–2012	11	9, 17, 25	Y, Y	N

The S2S model resolution is 1.5° × 1.5° and ACCESS-S1 is 0.05° × 0.05°. All forecasts extend through to the end of the flood event (i.e., 6 February 2019), even for models with the shortest time range of 0–32 days (i.e., ECCC and ISAC-CNR) which finish on the 11 February for a 10 January initialisation.

Accumulated daily precipitation was converted into daily precipitation totals, while near-surface wind-speeds were determined from 10 m *U* and *V* winds. For maximum and minimum temperatures, 6-hourly 2 m data were averaged to produce daily near-surface mean temperatures. Across the S2S models, we identified which ensemble forecasts skilfully predicted the relatively high probability of extreme livestock chill conditions during the February 2019 event based on what they deemed extreme in their own hindcasts (see “S2S prediction of the stalled MJO” Section). The comparison to a model's own hindcast is necessary, as opposed to absolute wind chill conditions, because a subset of S2S models, including ECMWF, Météo-France (CNRM), NCEP and POAMA, broadly under-forecast the total rain associated with the floods, which was record-breaking at multiple weather stations^1^.

An additional assessment of the forecasted average livestock chill conditions during the week of 31 January–6 February 2019 at lead week 1 (Suppl. Fig. 7) shows that the S2S models substantially underestimated the extreme conditions 1 week prior to the event, with only two models (UKMO and ISAC-CNR) forecasting values of 900 kJ/m^2^/h in the Gulf region, whereas observed values exceeded 1000 kJ/m^2^/h. The lead week 0 forecasts (Suppl. Fig. 8) show strong improvements in the structure and magnitude of the chill conditions, although three S2S model forecasts (CMA, ECCC, Météo-France) place the peak chill region off-centre from the Gulf region. The fact that the S2S model outputs are raw and not calibrated against observations likely contributed to the underestimates of rainfall^1^ and livestock chill conditions in the lead week 1 forecasts.

We also used available MJO indices from nine S2S models (Table 1), to verify if the stalled MJO was predictable, extending the analysis of recent studies on the extreme event^1,15^. One hypothesis is that the MJO stalling in phases 6 and 7 (i.e., western Pacific) was associated with the near-stationary tropical low and hence extreme weather conditions^1^. Unfortunately, the MJO index predictions were unavailable^20^ from ACCESS-S1 during early 2019. We also made use of the S2S model's daily mean sea level pressure (MSLP) to compare the predictions of the large-scale surface circulation between model members that better predicted the MJO to stall. As a reference point, we determined daily MSLP anomalies from NCEP-NCAR Reanalysis 1 over the extreme event period using a long-term 1981–2010 climatology^44^.

### Evaluating the extreme conditions

When comparing ACCESS-S1 and S2S forecasts of the probability of extreme conditions during the flood event, we first defined what was considered 'extreme'. Aware that a livestock chill value of > 1000 kJ/m^2^/h may be considered high for southern Australia, we deemed any values above the multi-year (e.g., long-term) calendar day 90th percentile threshold to be considered extreme. In the same way that studies have selected heat wave thresholds^45^, the multi-year calendar day 90th percentile uses a 15-day window across the observational (1971–2018) or hindcast (see Table 1) periods. The large sample sizes provided us with a suitable population for extreme events to be sampled from, while the 15-day moving window accounted for any seasonal cycle in temperatures and rainfall. We applied the same method for rainfall and winds, while for temperatures we calculated both the 10th (cold) and 90th (hot) percentiles. For the hindcasts we determined the multi-year extreme conditions (percentiles) for each hindcast start date, and we also combined each hindcast ensemble member beforehand. For example, for ACCESS-S1, with its 23 hindcast years (1990–2012) and 11-members, each calendar day 90th percentile was determined from 3795 days (15 days × 11 members × 23 hindcast years).

## Supplementary Information

Supplementary Information.

## Data Availability

The S2S forecasts and hindcasts are available at: https://apps.ecmwf.int/datasets/data/s2s-realtime-instantaneous-accum-ecmf. Forecast and hindcast data from ACCESS-S1 (now updated to ACCESS-S2) is available on request. Daily MSLP from NCEP-NCAR Reanalysis 1 is available for download at: https://psl.noaa.gov/data/gridded/data.ncep.reanalysis.surface.html.

## Code availability

The code used to generate the figures and Supplementary figures is available on request.
