# Supplementary material for: Multi-week prediction of livestock chill conditions associated with the northwest Queensland floods of February 2019
Source: Sci Rep. 2022 Apr 8;12:5907. doi: 10.1038/s41598-022-09666-z (PMC8993847; doi:10.1038/s41598-022-09666-z)
Supplement: Supplementary file 1 — Supplementary Information. [file 41598_2022_9666_MOESM1_ESM.docx]

Supplementary Material for:

**Cowan et al. : Multi-week prediction of livestock chill conditions associated with the northwest Queensland floods of February 2019**

Supplementary Material includes:

Suppl. Figure 1: Daily livestock chill conditions during the northwest Queensland flood event.

Suppl. Figure 2: Lead week 1 forecast of the chances for extreme livestock chill conditions from ACCESS-S1 and ten S2S forecast systems, compared to the observations.

Suppl. Figure 3: Lead week 1 forecast of the chances of extreme precipitation from ACCESS-S1 and ten S2S forecast systems.

Suppl. Figure 4: Lead week 1 forecast of the chances of extreme cold maximum temperatures from ACCESS-S1 and ten S2S forecast systems.

Suppl. Figure 5: Lead week 1 forecast of the chances of extreme wind speeds from ACCESS-S1 and ten S2S forecast systems.

Suppl. Figure 6: Time evolution of MJO predictions for six S2S model forecast ensembles that predicted extreme livestock chill conditions, initialised on 24 January (lead week 1 forecast).

Suppl. Figure 7: Lead week 1 forecast of the average livestock chill conditions from ACCESS-S1 and ten S2S forecast systems, compared to the observations.

Suppl. Figure 8: Lead week 0 forecast of the average livestock chill conditions from ACCESS-S1 and ten S2S forecast systems, compared to the observations.


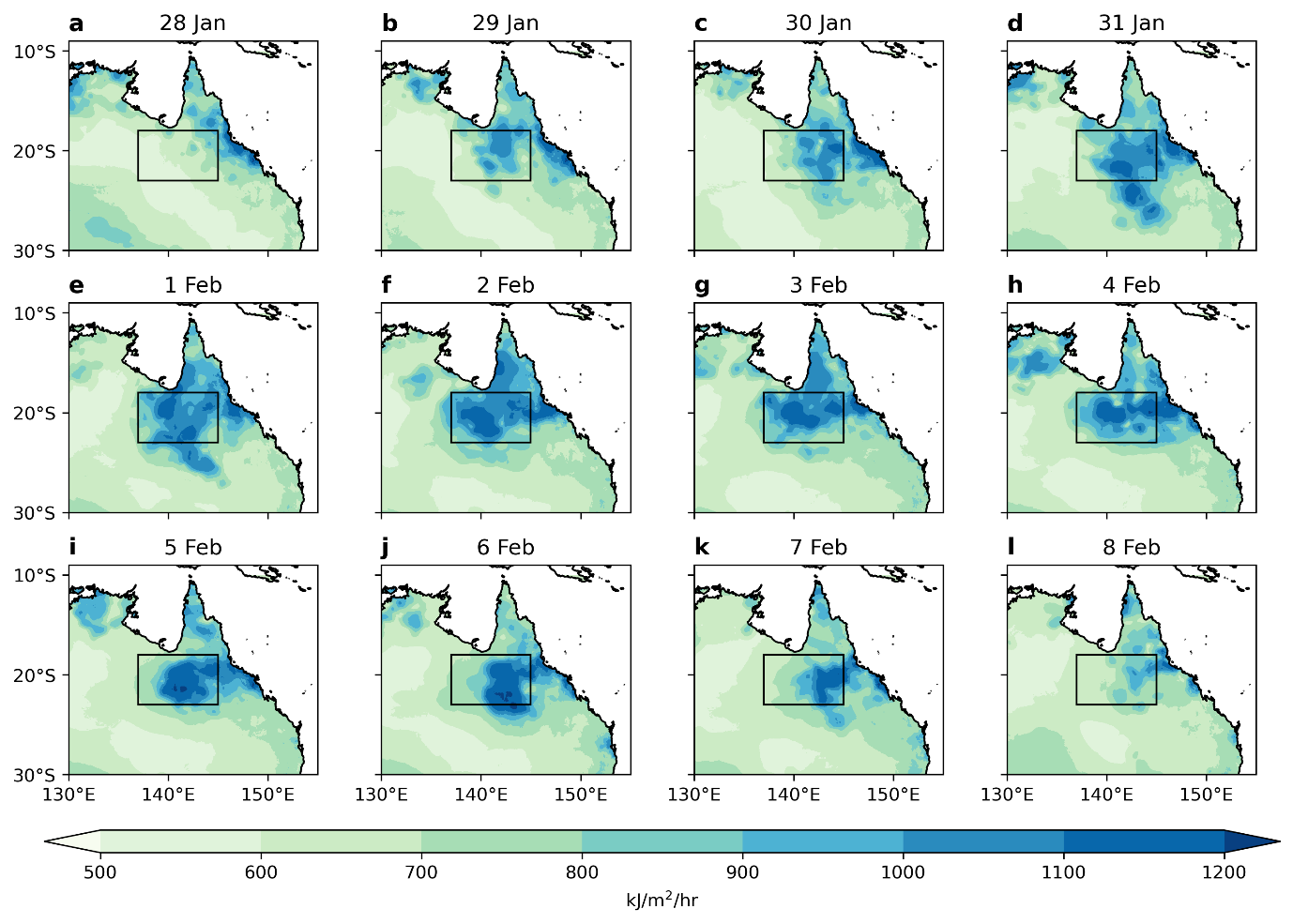


**Suppl. Figure 1: Daily livestock chill conditions during the northwest Queensland flood event.** The maps show the daily conditions from 28 January through to 8 February 2019. The day with the most extreme conditions averaged over the Gulf region was 2 February with an average of 1005 kJ/m^2^/hr, although the livestock chill conditions reached 1221 kJ/m^2^/hr in the southern part of the region on 5 February.

**
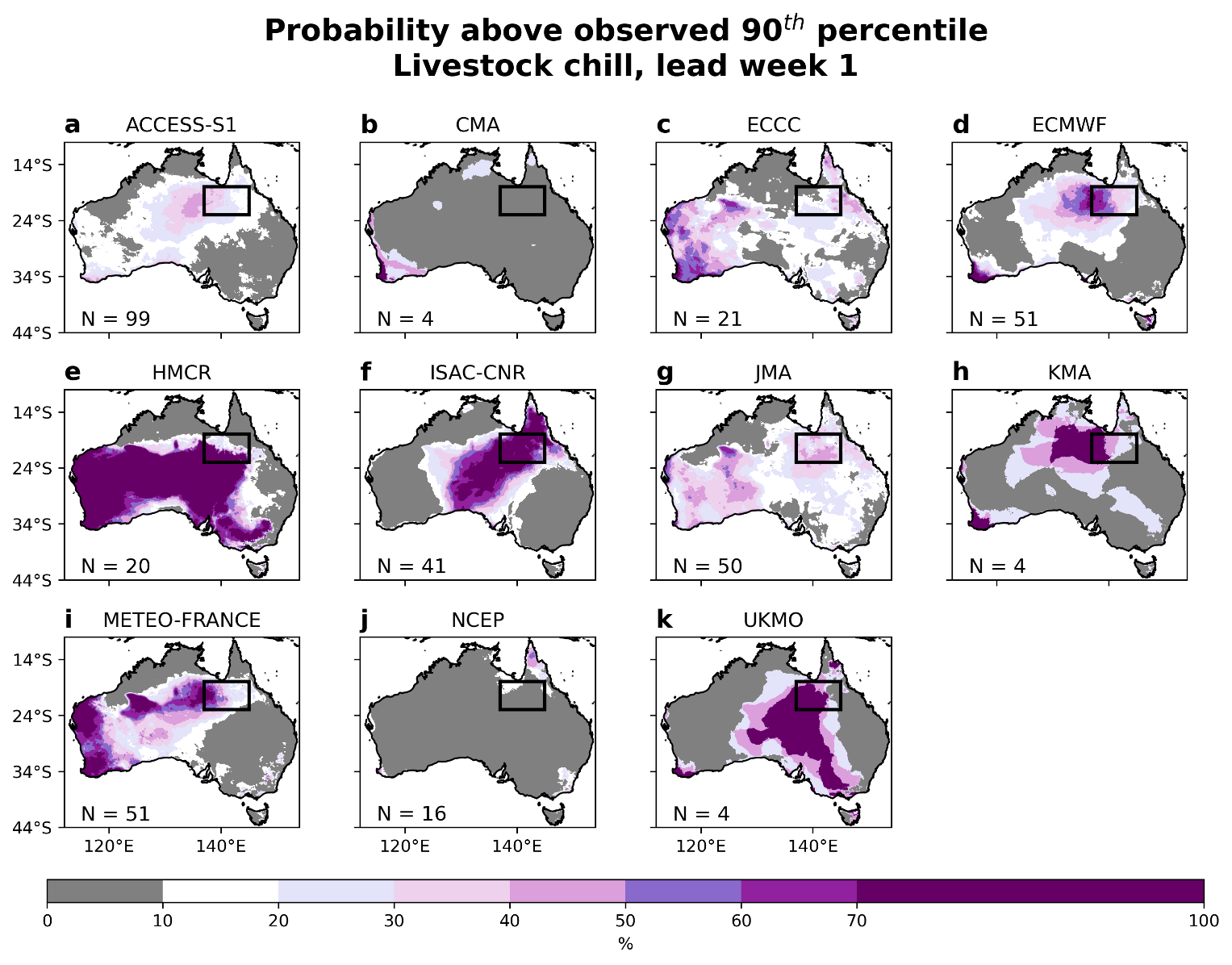
**

**Suppl. Figure 2: Lead week 1 forecast of the chances for extreme livestock chill conditions from ACCESS-S1 and ten S2S forecast systems, compared to the observations.** As in Figure 4, but for forecasts above the observed multi-year daily 90^th^ percentile determined over 1971-2018. All S2S models are first bilinearly regridded to a 0.05° resolution to match the AWAP gridded observations.

**
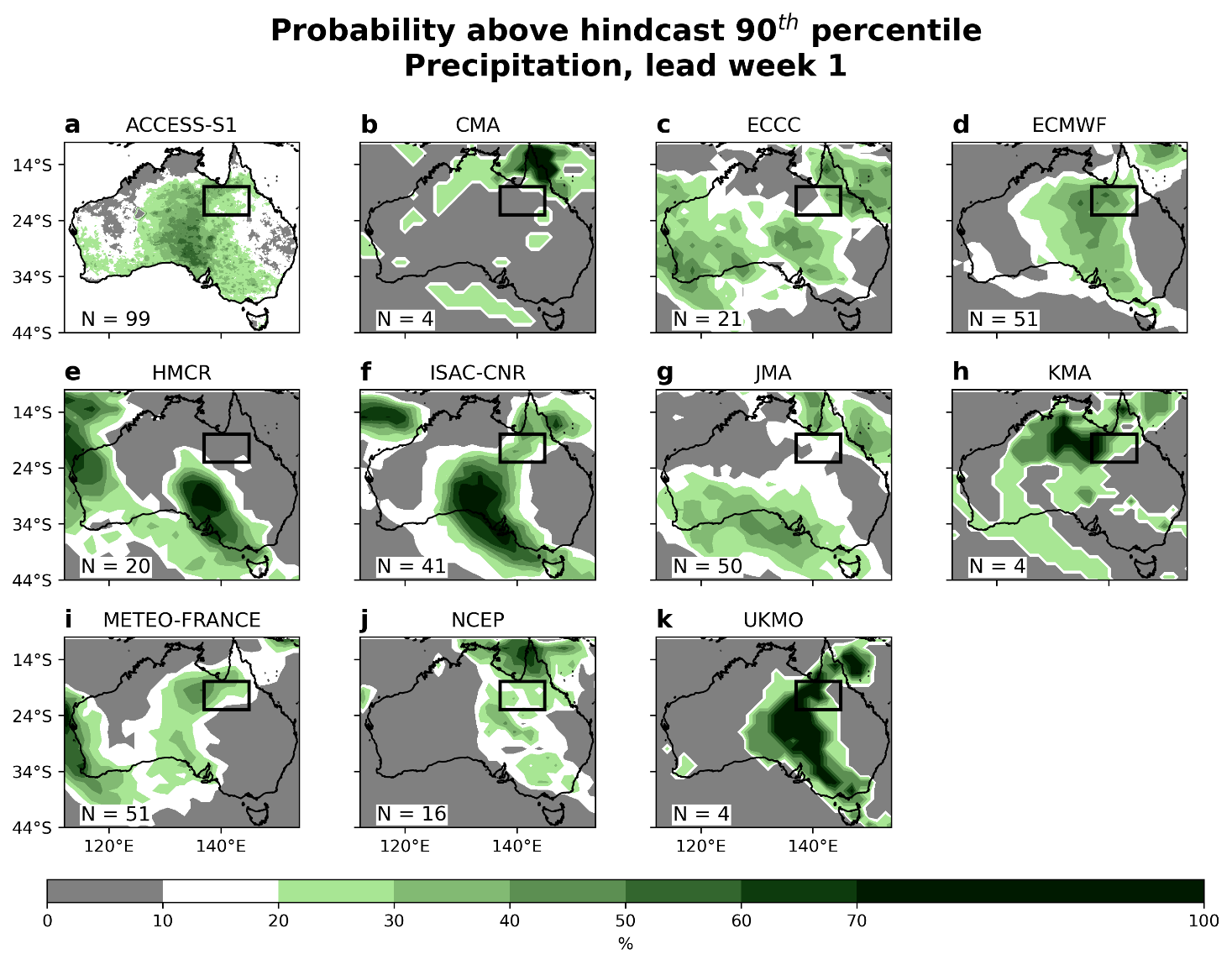
**

**Suppl. Figure 3: Lead week 1 forecast of the chances of extreme precipitation from ACCESS-S1 and ten S2S forecast systems.** As in Figure 4, but for forecasts of daily precipitation above each model's own hindcast multi-year daily 90^th^ percentile.

**
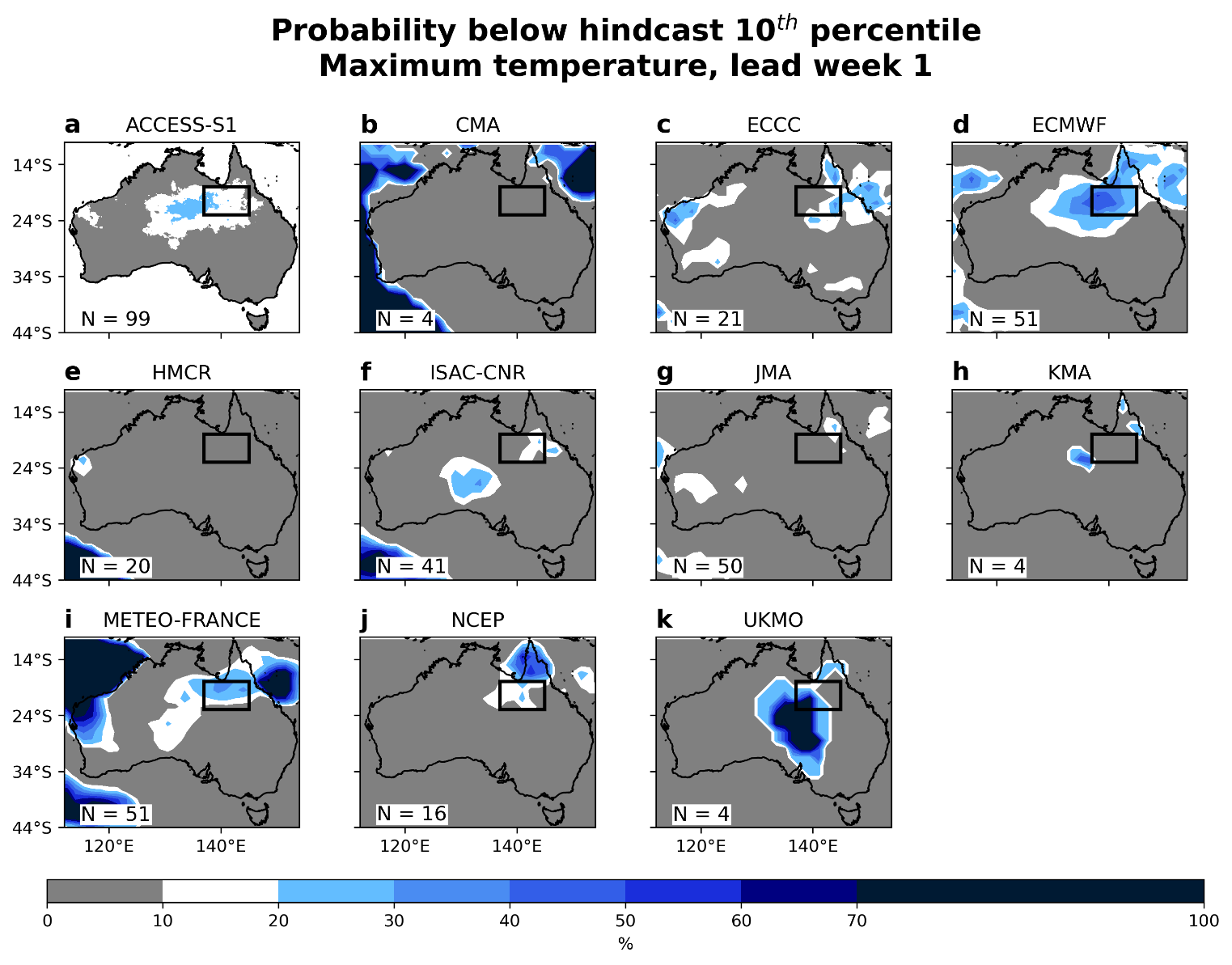
**

**Suppl. Figure 4: Lead week 1 forecast of the chances of extreme cold maximum temperatures from ACCESS-S1 and ten S2S forecast systems.** As in Figure 4, but for forecasts of daily maximum temperature below each model's own hindcast multi-year daily 10^th^ percentile.

**
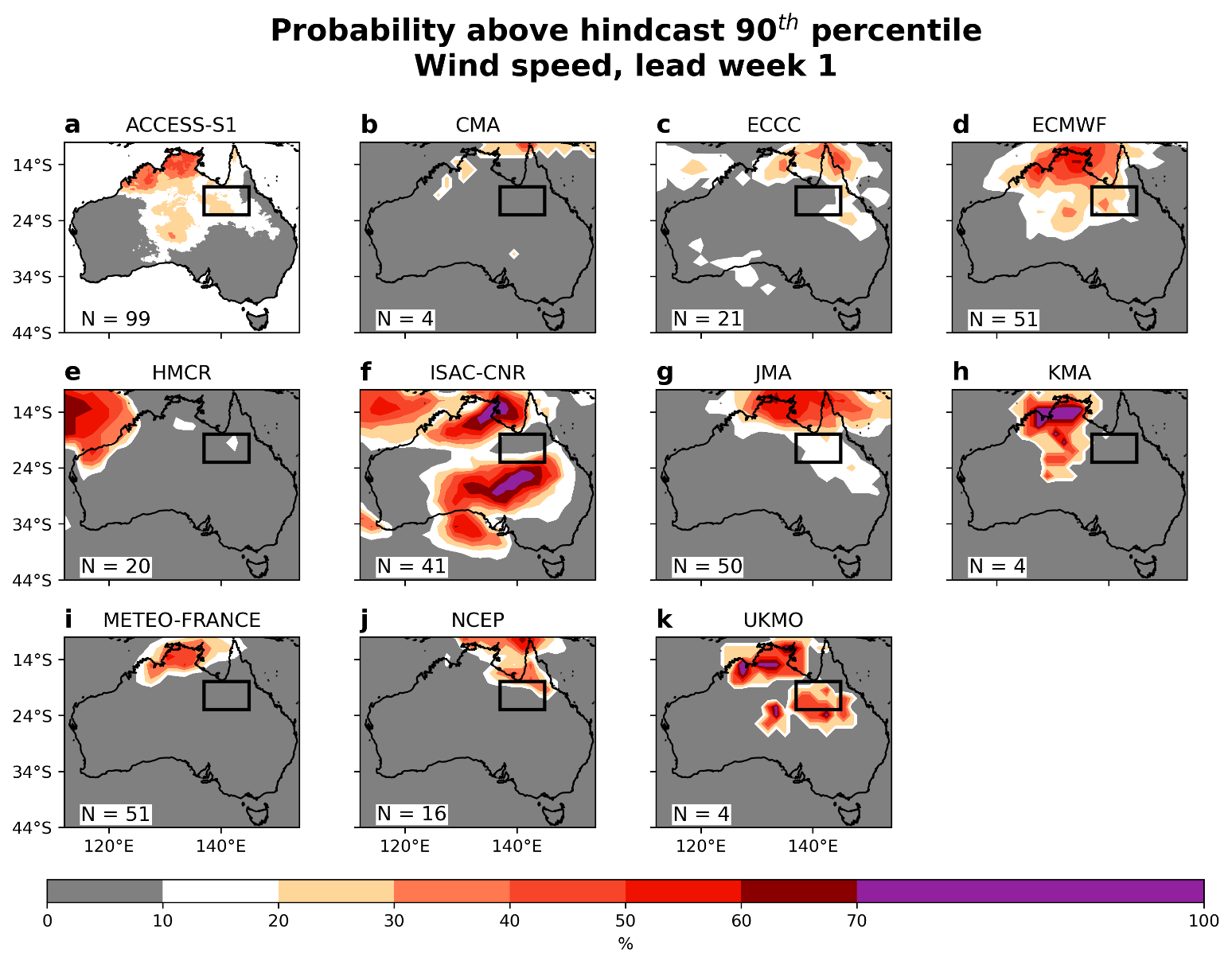
**

**Suppl. Figure 5: Lead week 1 forecast of the chances of extreme wind speeds from ACCESS-S1 and ten S2S forecast systems.** As in Figure 4, but for forecasts of daily wind speed above each model's own hindcast multi-year daily 90^th^ percentile.


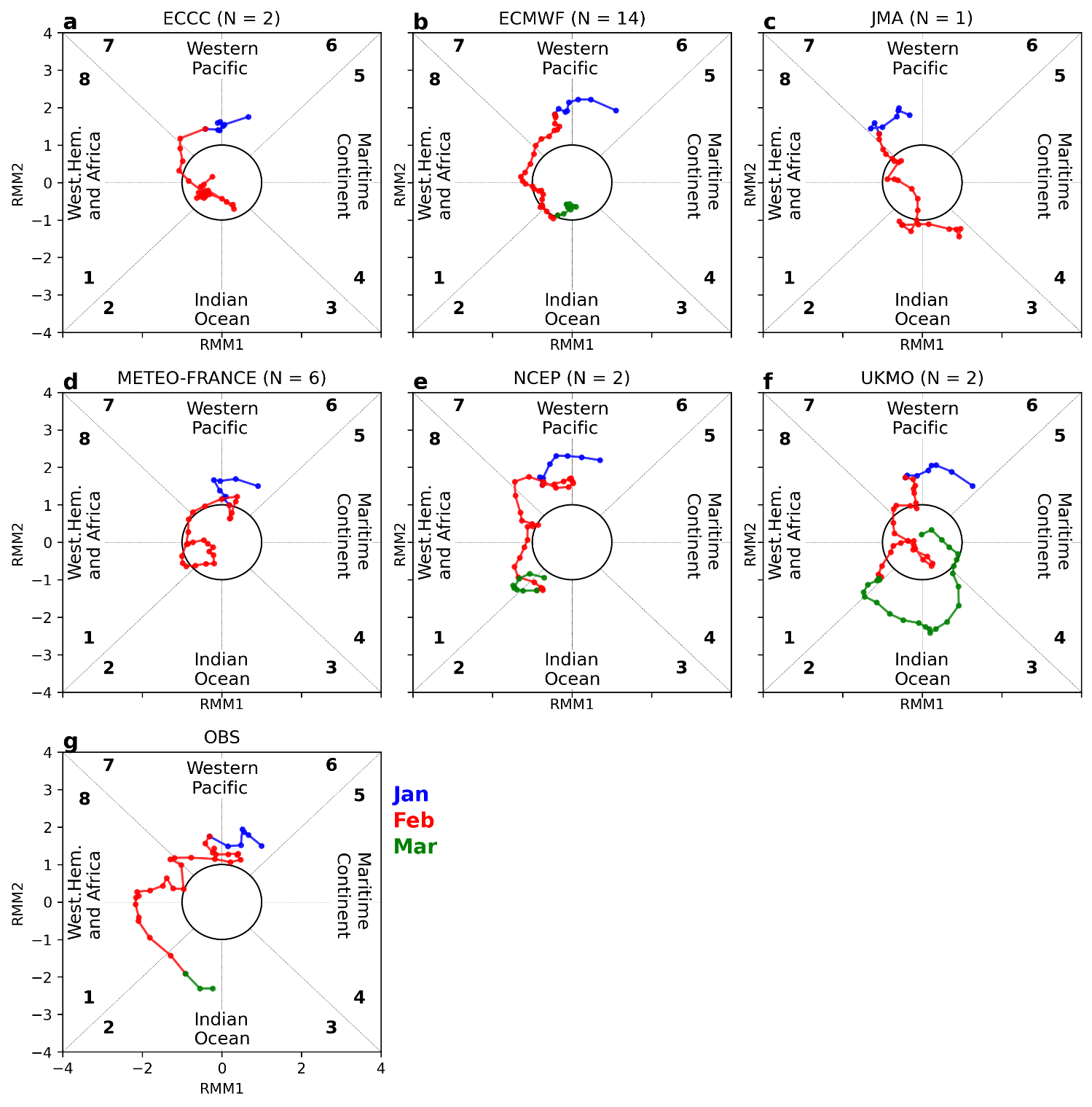


**Suppl. Figure 6: Time evolution of MJO predictions for six S2S model forecast ensembles that predicted extreme livestock chill conditions, initialised on 24 January (lead week 1 forecast).** Shown are the evolutions of MJO predictions (ensemble means and single members) from (a-f) model experiments where the livestock chill predictions showed extreme conditions (over the peak flood week) for more than 60% of the inland Gulf region. Also shown are (g) observations. Please note, JMA is initialised on 23 January, hence its first date is 24 January. Despite not all the MJO forecasts extending to March, all cover the early February flood event. The CMA, HMCR and ISAC-CNR models are not shown as they did not predict extreme wind chill conditions in over 60% of the inland Gulf region in any of their ensemble members.


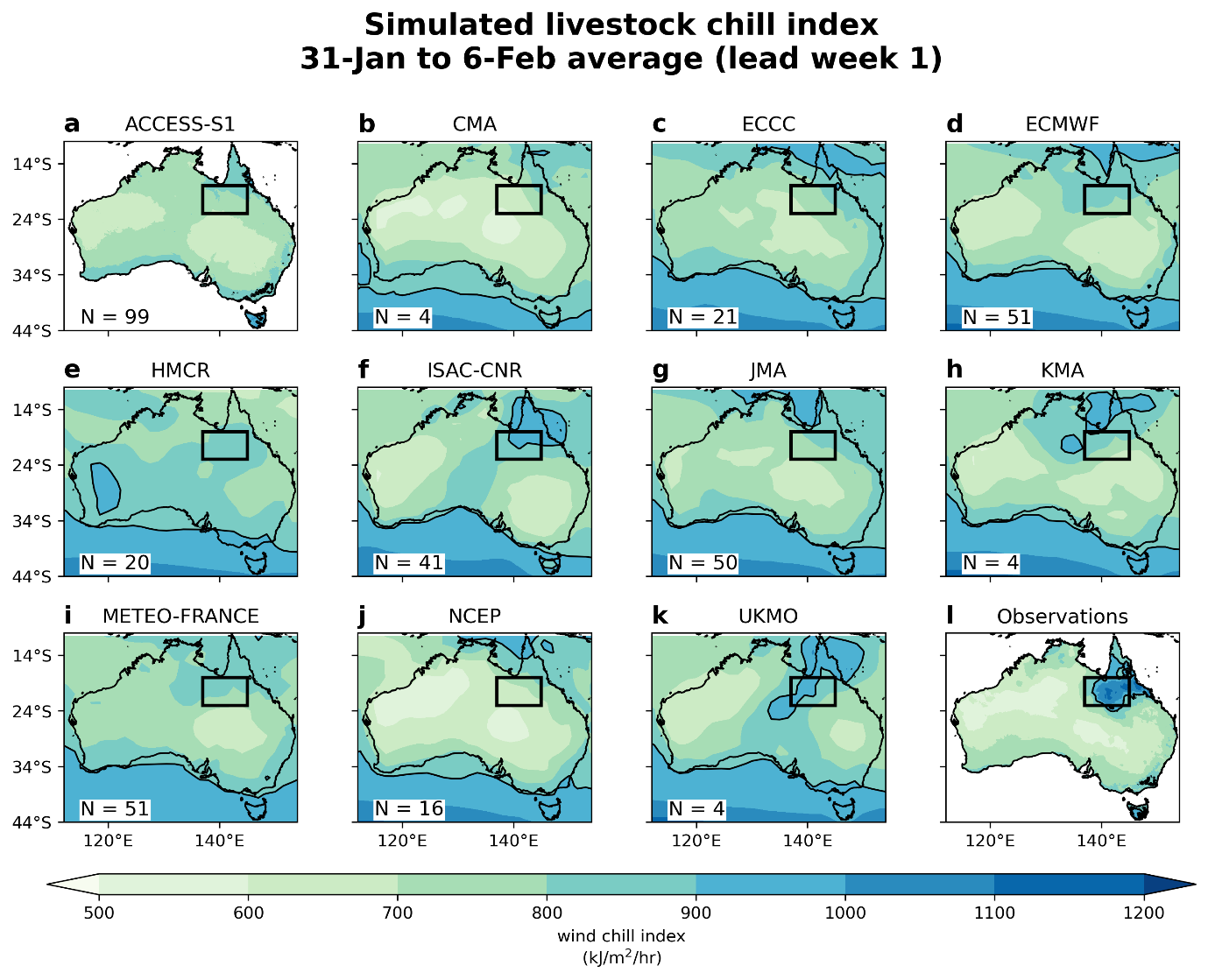


**Suppl. Figure 7: Lead week 1 forecast of the average livestock chill conditions for the week 31 January to 6 February 2019, from ACCESS-S1 and ten S2S forecast systems, compared to the observations.** The 900 kJ/m^2^/hr contour line is highlighted in the (a-k) forecasts and (l) observations to highlight that the models underestimate the chill conditions.


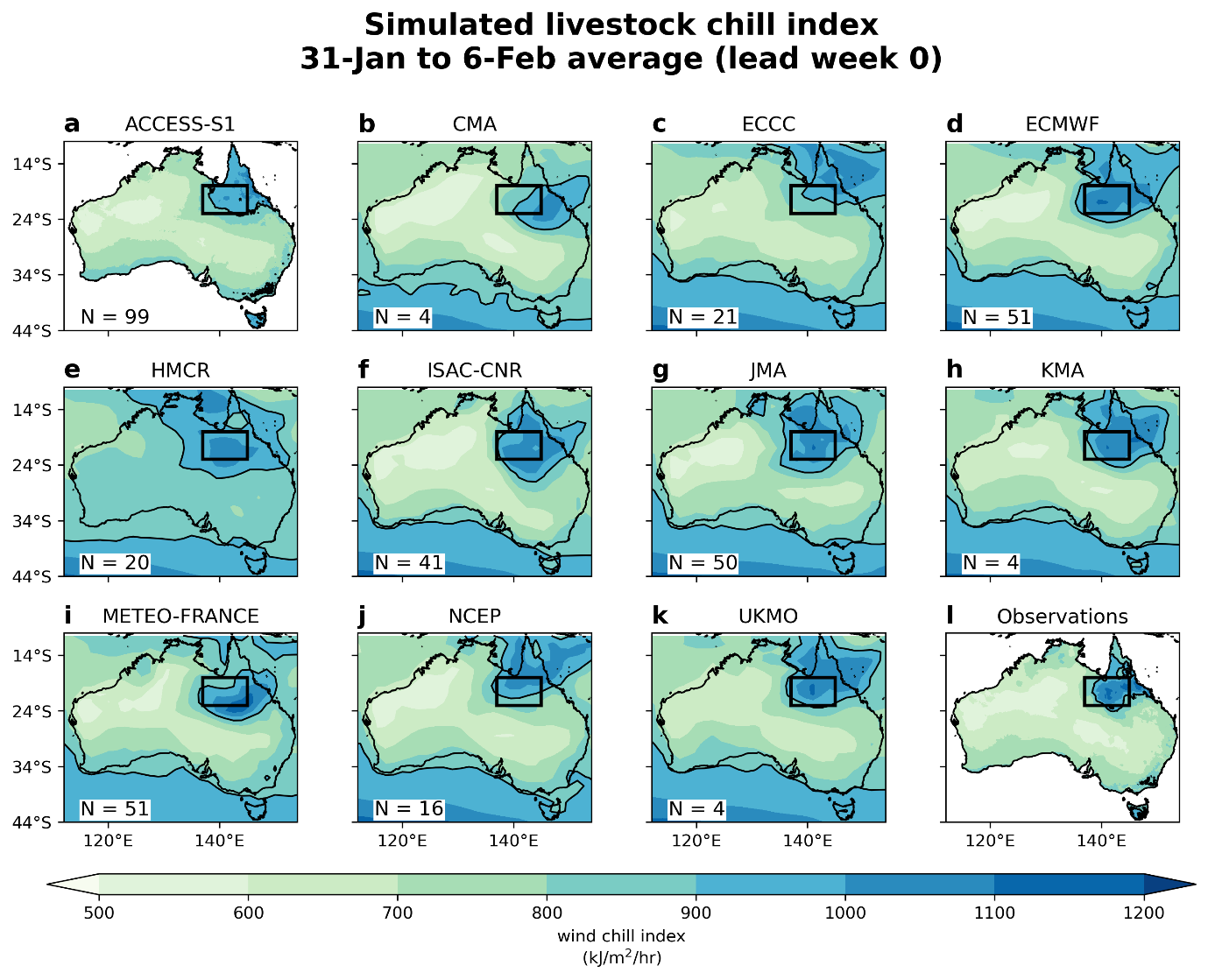


**Suppl. Figure 8: Lead week 0 forecast of the average livestock chill conditions for the week 31 January to 6 February 2019, from ACCESS-S1 and ten S2S forecast systems, compared to the observations.** The 900 kJ/m^2^/hr contour line is highlighted in the (a-k) forecasts and (l) observations.
